# Long-Term Effectiveness of Spinal Cord Stimulation Beyond 24 Months: A PRISMA-ScR-Informed Scoping Review

**DOI:** 10.3390/jcm15103939

**Published:** 2026-05-20

**Authors:** Jakub Wiśniewski, Mateusz Szczupak, Paweł Jan Winklewski, Anna Barbara Marcinkowska

**Affiliations:** 1 Department of Neurosurgery, Nicolaus Copernicus Hospital, 80-803 Gdansk, Poland; 2 Department of Anaesthesiology and Intensive Therapy, Nicolaus Copernicus Hospital, 80-803 Gdansk, Poland; 3 Department of Neurophysiology, Neuropsychology and Neuroinformatics, Medical University of Gdansk, 80-210 Gdansk, Poland; pawelwinklewski@wp.pl; 4 2nd Department of Radiology, Medical University of Gdansk, 80-210 Gdansk, Poland; 5 Applied Cognitive Neuroscience Lab, Department of Neurophysiology, Neuropsychology and Neuroinformatics, Medical University of Gdansk, 80-210 Gdansk, Poland

**Keywords:** spinal cord stimulation, neuromodulation, chronic pain, long-term outcomes, durability, failed back surgery syndrome, persistent spinal pain syndrome, painful diabetic neuropathy, nonsurgical refractory back pain, closed-loop stimulation, scoping review, PRISMA-ScR, evidence mapping

## Abstract

**Background/Objectives:** Spinal cord stimulation (SCS) is an established therapy for chronic refractory pain, but its clinical value depends on whether benefit persists beyond the early post-implant period. Although short-term SCS studies are abundant, reports with follow-up of 24 months or longer are dispersed, methodologically heterogeneous, and difficult to interpret across indications and stimulation platforms. This scoping review aimed to map the clinical literature reporting SCS outcomes at ≥24 months, characterize the represented populations and modalities, summarize the long-term outcome domains assessed, and identify major methodological and clinical gaps in the evidence base. **Methods:** This PRISMA-ScR-informed scoping review applied a Population–Concept–Context framework. PubMed/MEDLINE and Scopus were searched through April 2026, yielding 6866 records before deduplication. Following staged title/abstract screening, iterative full-text retrieval, and the reconciliation of overlapping publications, 292 unique full-text reports were assessed for eligibility. Studies reporting original clinical SCS data with extractable outcomes at ≥24 months were included. No meta-analysis or formal GRADE assessment was undertaken, as the objective was evidence mapping rather than pooled effect estimation. **Results:** The final evidence map comprised 65 unique reports representing a cumulative report-level population of 11,518 participants across non-overlapping cohorts. The literature was dominated by non-randomized evidence (55 observational reports; 10 randomized or randomized-derived). The most frequent indication was mixed chronic pain (30/65; 46.2%), followed by failed back surgery syndrome/persistent spinal pain syndrome (FBSS/PSPS; 16/65; 24.6%), chronic back and/or leg pain (6/65; 9.2%), complex regional pain syndrome (CRPS; 5/65; 7.7%), and painful diabetic neuropathy (PDN; 4/65; 6.2%). Most reports involved conventional or unspecified SCS (47/65; 72.3%), with smaller contemporary clusters for 10 kHz high-frequency SCS and ECAP-controlled closed-loop SCS. The most frequently reported outcome domains were pain durability, function and quality of life, device-related outcomes, and opioid use. At a descriptive level, the literature more often supported persistence of benefit than complete erosion of effect, particularly in spinal pain populations and in contemporary PDN and closed-loop SCS cohorts. Interpretation was constrained by outcome heterogeneity, cohort overlap, mixed indication categories, and inconsistent opioid and device-maintenance reporting. **Conclusions:** The long-term SCS literature supports the view that durable benefit is achievable in a substantial patient subset, particularly in FBSS/PSPS populations, and in more recent evidence, in PDN, nonsurgical refractory back pain, and closed-loop SCS cohorts. The evidence base remains heterogeneous and does not support a uniform certainty-ranked estimate across indications and technologies. Future studies should prioritize indication-specific cohorts, standardized multidomain outcome reporting, and transparent separation of unique cohorts from secondary analyses of the same clinical populations.

## 1. Introduction

Spinal cord stimulation (SCS) is an established neuromodulation therapy for chronic refractory pain, particularly persistent spinal pain after prior surgery, chronic back and leg pain, and selected neuropathic pain syndromes [1,2,3]. The historical evidence base was built largely in failed back surgery syndrome and related spinal pain populations, but more recent work has expanded the field toward painful diabetic neuropathy, nonsurgical refractory back pain, and physiologic closed-loop stimulation paradigms. At the same time, the technological landscape has diversified from conventional paresthesia-based systems to high-frequency, burst, and feedback-guided platforms, increasing both the therapeutic scope of SCS and the heterogeneity of the literature used to evaluate it [3,4,5,6,7].

Because SCS is intended as a durable implantable therapy rather than a short-course procedural intervention, its clinical value cannot be judged solely by trial success or early post-implant analgesia [1,2,8]. The clinically relevant question is whether benefit is sustained beyond the initial postoperative period and whether that benefit is accompanied by durable gains in function, health-related quality of life, and treatment stability, while maintaining an acceptable burden of revision, explantation, and late loss of efficacy [1,2,8,9]. This distinction is central in routine practice, where decisions regarding implantation, patient counselling, reimbursement, and long-term follow-up depend on the expected durability rather than on transient short-term improvement alone [1,2,8,9].

Durability is also relevant from a health-economic perspective. SCS is associated with substantial upfront costs related to trialing, implantation, hardware, programming, and follow-up, so its value depends heavily on the duration of maintained benefit [10,11]. In a randomized cost-utility analysis in failed back surgery syndrome, SCS was less expensive and more effective than reoperation over a mean follow-up of 3.1 years [10]. In a later model-based economic evaluation using SENZA trial data, 10 kHz SCS was reported to be cost-saving and cost-effective compared with low-frequency SCS over a 15-year horizon [11]. These findings strengthen the rationale for focusing on long-term outcomes, although the available economic evidence arises from specific clinical comparisons and may not apply uniformly across indications and stimulation platforms across all indications and SCS technologies [10,11].

Despite the importance of long-term effectiveness, much of the published SCS literature remains concentrated on short-term outcomes, commonly within 6 to 12 months after implantation [4,5,6]. Such studies are valuable for demonstrating early efficacy and feasibility, but they do not fully characterize persistence of benefit. They also provide only limited information on the longer-term trajectory of function and quality of life, the evolution of opioid use, and the burden of reprogramming, revision, and device discontinuation [4,5,6,8,9]. More mature follow-up data are now available for some populations, including painful diabetic neuropathy, nonsurgical refractory back pain, and closed-loop SCS cohorts, but these reports remain dispersed across diverse designs, indications, and outcome frameworks [3,5,6,7,12,13]. The literature beyond 24 months is therefore clinically important but methodologically fragmented.

Reports with follow-up beyond 24 months vary substantially in patient selection, indication mix, stimulation modality, outcome definitions, and the depth of reporting for pain durability, function, quality of life, revision, explantation, complications, and opioid use [1,2,3,5,6,7,8,9,12,13]. A focused synthesis of this longer-term evidence base is therefore needed, not to infer a single pooled treatment effect, but to clarify what is currently known about sustained effectiveness, where the evidence is strongest, and where important uncertainties remain. The 24-month threshold was selected a priori as the minimum follow-up horizon beyond which the post-implant adaptation phase can reasonably be considered complete and treatment response can be interpreted as sustained rather than early. The aim of this PRISMA-ScR-informed scoping review was to map the available clinical literature reporting SCS outcomes at 24 months or longer, describe the represented populations and stimulation modalities, summarize the long-term outcome domains assessed, and identify the principal methodological and clinical gaps in the current evidence base.

## 2. Materials and Methods

### 2.1. Study Design and Reporting Framework

This study was conducted as a scoping review informed by the methodological framework of Arksey and O’Malley, subsequent refinements proposed by Levac et al., and current Joanna Briggs Institute (JBI) guidance for scoping reviews [14,15,16]. Reporting was structured in accordance with the PRISMA extension for scoping reviews (PRISMA-ScR) [16]. The objective of the review was not to estimate a pooled treatment effect, but to map the longer-term SCS evidence base, describe the clinical populations and technologies represented, summarize the outcome domains reported beyond 24 months, and identify methodological gaps relevant to future research [15,16].

No prospective protocol was registered before initiation of the project. This represents an important methodological limitation. To improve transparency, the final review question, eligibility criteria, search framework, screening logic, and data-charting fields were fixed before preparation of the final synthesis and are reported explicitly in the present manuscript and Supplementary Materials. In the absence of prospective registration, the final search strings, staged selection logic, extraction fields, and report-level decisions are fully reproduced in the Supplementary Materials.

### 2.2. Review Question and Eligibility Framework

The review question was structured according to the Population–Concept–Context framework recommended for scoping reviews [15,16].

Population: Adults treated with spinal cord stimulation for chronic pain.

Concept: Long-term clinical effectiveness and durability of treatment effect, defined a priori as outcomes reported at 24 months or longer after implantation or after initiation of long-term therapy.

Context: Clinical studies, registries, prospective cohorts, retrospective cohorts, randomized trials, long-term follow-up reports, and secondary analyses of clinical trials or registries.

Studies were eligible for inclusion if they met all of the following criteria: (1) reported original clinical data on patients treated with SCS; (2) included extractable outcomes at ≥24 months; (3) evaluated at least one long-term clinical outcome domain, including pain relief, responder status, durability of effect, function, disability, quality of life, opioid use, explantation, revision, or complications; and (4) were indexed in PubMed/MEDLINE or Scopus and available in full text.

The following were excluded from the final synthesis: (1) single-patient case reports; (2) small technical or anecdotal case series without extractable group-level outcomes at ≥24 months; (3) reviews, commentaries, editorials, and protocol-only papers; (4) mechanistic, programming-only, technical feasibility, or peri-implant utilization studies without relevant long-term clinical outcomes; (5) studies reporting outcomes only below 24 months; (6) salvage or adjunct-treatment studies in which SCS was not the primary long-term therapeutic exposure of interest; and (7) papers without sufficient clinical detail to allow eligibility confirmation or data charting.

When multiple publications arose from the same clinical program, trial, or cohort, all reports were retained bibliographically, but cohort overlap was examined during charting and interpretation. The final narrative synthesis emphasized unique long-term report contributions rather than naive aggregation of all report-level patient counts.

### 2.3. Information Sources and Search Strategy

The literature search was performed in PubMed/MEDLINE and Scopus, selected to capture both indexed biomedical literature and a broad multidisciplinary citation base. Search development was pragmatic and iterative, reflecting the staged workflow of the project. The search logic combined terms for spinal cord stimulation with terms capturing long-term follow-up, durability, outcomes, and chronic pain indications. The exact operational search strings used in PubMed and Scopus are reproduced verbatim in Supplementary Table S1. Search terms were applied to title and abstract fields; MeSH indexing was not used as a primary search layer in order to maintain consistency between PubMed and Scopus query logic.

Searches were executed and export files were finalized through April 2026. The PubMed search returned 3188 records and the Scopus search returned 3678 records, for a total of 6866 records before cross-database reconciliation. Searches were designed to maximize sensitivity for long-term clinical reports rather than to target a single indication or stimulation modality. No date restrictions were imposed at the conceptual search stage.

Because the project evolved in a staged, practice-based manner and full-text acquisition occurred iteratively over time, the search and screening process did not proceed as a single database export followed by one locked deduplication event. Instead, records were reviewed in sequential screening waves and later reconciled against the full-text corpus obtained during the project. This workflow was preserved and is reported transparently below.

### 2.4. Selection Process

Selection proceeded in stages. From the broader combined database yield, a manually reviewed title/abstract screening set of 604 records was assembled. This subset was derived after the reconciliation of database exports and a retrospectively defined relevance-based pre-screening framework intended to remove records that were clearly outside the scope of the review. This pre-screening step was performed independently by two reviewers working from exported bibliographic fields (title, abstract, and source journal) without access to full-text documents; records were excluded at this stage only when both reviewers agreed that the exclusion criteria were unequivocally met on the basis of available export-level information alone. At the pre-screening stage, records were excluded if the title/abstract information unequivocally indicated one or more of the following: (1) non-clinical record types, including reviews, editorials, commentaries, conference abstracts without full-text availability, and protocol-only papers; (2) intervention outside scope, including non-SCS neuromodulation modalities (e.g., DBS, PNS, tDCS) with no SCS arm, pharmacological or surgical studies without SCS component, and basic science or animal studies; (3) population outside scope, defined as pediatric populations, non-pain indications, or acute/perioperative pain; and (4) follow-up clearly below 24 months when explicitly stated in the title or abstract. When eligibility on any of these criteria was not clearly negated from the title/abstract information alone, records were retained for formal screening. Because this framework was defined retrospectively rather than prospectively protocolized, the possibility that some potentially eligible studies were excluded before formal screening cannot be fully eliminated and is acknowledged as a limitation.

Of the 6866 records identified through database searching, 6262 were excluded at the pre-screening stage and 604 were retained for formal title/abstract review. The principal exclusion categories at pre-screening were, in approximate order of frequency: non-clinical record types (reviews, editorials, commentaries, conference abstracts without full-text availability, and protocol-only papers; approximately 2950 records); intervention outside scope (non-SCS neuromodulation modalities, pharmacological or surgical studies without an SCS component, and basic science or animal studies; approximately 1900 records); population outside scope (pediatric populations, non-pain indications, and acute or perioperative pain; approximately 880 records); and follow-up clearly below 24 months when explicitly stated in the title or abstract (approximately 530 records). The remainder reflected near-duplicate records and indexing artifacts identified during reconciliation between PubMed and Scopus exports. A structured numerical breakdown of pre-screening exclusion categories is provided in Supplementary Table S2A. Inter-reviewer agreement at pre-screening was high, reflecting the fact that records excluded at this stage met clearly unequivocal export-level criteria; in the small number of disagreements, records were retained for formal title/abstract review rather than excluded, in order to favor sensitivity over specificity at this stage. Title/abstract screening of the 604-record set was conducted in sequential waves by two reviewers working independently using Microsoft Excel (version 16.109; Microsoft Corporation, Redmond, WA, USA), with disagreements resolved by discussion, and when needed, adjudication by a third reviewer. In the first title/abstract pass, 277 records were classified as potentially relevant, 80 as uncertain, and 247 as excluded. A second-pass review was then performed on the 357 retained or uncertain records, yielding 152 records considered potentially eligible, 112 still uncertain, and 93 excluded. This process generated a practical candidate pool of 264 records for full-text retrieval.

During full-text acquisition and reconciliation, an additional 28 full-text reports were identified through project-level citation chasing, DOI-targeted retrieval, linked follow-up publications from already retained trials or cohorts, and the reconciliation of mature long-term reports not fully captured within the initial manually screened subset. This explains the difference between the 264 post-screening candidate records and the 292 unique full texts ultimately screened in full.

Across the entire workflow, 292 unique full-text reports were assessed for eligibility. Full-text review was performed independently by two reviewers, with disagreements resolved by consensus, and where necessary, by arbitration from a third reviewer. Of these 292 reports, 202 were excluded after full-text assessment and 90 were initially retained within the uploaded full-text corpus. After the reconciliation of overlapping publications, removal of non-eligible duplicate report contributions, and restriction to reports with extractable long-term outcomes aligned with the final review question, the final synthesis included 65 unique reports with outcomes at ≥24 months.

**Figure 1:**
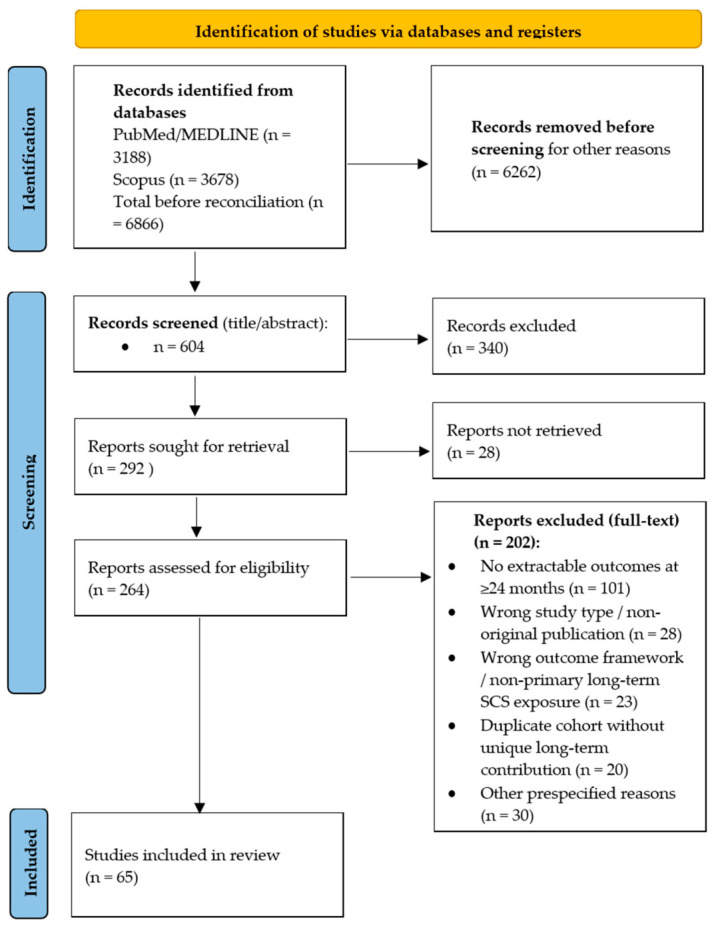
PRISMA-ScR flow diagram summarizing the staged study selection, full-text eligibility assessment, and final inclusion of reports with extractable outcomes at ≥24 months.

The most common reason for exclusion at full-text stage was the absence of extractable outcomes beyond 24 months. Other recurring reasons included wrong study type, non-eligible outcome framework, duplicate cohort reporting without unique long-term contribution, and insufficient clinical detail for charting. A PRISMA-ScR flow diagram summarizing the staged selection process is provided in Figure 1, and full-text exclusion categories are summarized in Supplementary Table S2.

### 2.5. Screening Personnel and Resolution of Disagreements

Title/abstract screening, full-text eligibility assessment, and final report reconciliation were conducted using a double-reviewer process. At each stage, two reviewers assessed records independently. Disagreements were resolved through discussion and consensus. When consensus was not reached, a third reviewer served as an arbiter. This approach was used to improve consistency in eligibility decisions across the staged workflow and to reduce the risk of the erroneous exclusion of clinically relevant long-term reports.

### 2.6. Unit of Analysis and Handling of Overlapping Reports

The report was the unit of screening, because the same clinical program or cohort often generated several publications focused on different outcome domains or follow-up windows. However, the evidence map required a higher level of interpretation than simple report counting. Accordingly, reports were examined for probable cohort overlap on the basis of trial name, center, recruitment period, indication, sample size, stimulation modality, and author group.

When multiple papers described the same mature cohort, they were retained in the bibliography but treated cautiously during synthesis. Report-level patient numbers were tabulated for transparency, but they were not interpreted as equivalent to unique individuals. For that reason, the cumulative patient total is reported explicitly as a report-level population count, not as a unique-participant denominator.

### 2.7. Data Charting

Data were charted using a prespecified extraction framework developed for this review in Microsoft Excel (version 16.109; Microsoft Corporation, Redmond, WA, USA). Charting was performed by two reviewers, with independent extraction followed by a comparison and reconciliation of entries. Any discrepancies were resolved through discussion, and when required, review by a third author. Earlier extraction fields were retained where possible, and additional interpretive fields were introduced only when necessary to improve consistency across eras and report types.

The charting fields included: bibliographic information; study design; country or region; clinical indication; SCS modality or platform; comparator, if any; total and implanted sample size; longest reported follow-up; long-term outcome domains reported; summary of pain outcomes; summary of functional and quality-of-life outcomes; opioid-related outcomes; revision, explanation, and complication reporting; overlap flag and notes relevant to interpretation; and declared industry sponsorship and/or conflicts of interest, when reported.

When the same cohort generated several publications, charting emphasized the most mature or most outcome-informative report while preserving linked references for traceability. The final extraction tables are presented in the Supplementary Materials.

### 2.8. Critical Appraisal and Certainty Assessment

Formal critical appraisal is optional rather than mandatory in scoping review methodology [15,16]. In the present review, no design-specific risk-of-bias tool was used to exclude studies, and no formal GRADE certainty-of-evidence assessment was undertaken. This was because the primary objective was evidence mapping rather than comparative effectiveness estimation. Established tools that would be appropriate for a future comparative effectiveness synthesis in this field include the Cochrane Risk of Bias 2 (RoB 2) tool for randomized trials [17], ROBINS-I for non-randomized studies of interventions [18], and the Newcastle–Ottawa Scale for cohort studies [19]; standardized outcome reporting in chronic pain trials is addressed by the IMMPACT recommendations [20]. These tools were not applied here because their primary purpose is to inform effect-estimation and certainty-of-evidence judgments rather than evidence mapping, but they are noted as relevant references for subsequent systematic reviews and meta-analyses of long-term SCS effectiveness.

Nevertheless, to avoid overinterpretation, the following design features were explicitly charted and considered in the synthesis: randomized versus non-randomized design; prospective versus retrospective data collection; multicenter versus single-center setting; report maturity and follow-up duration; probable cohort overlap; completeness of long-term outcome reporting; degree of clinical and technological heterogeneity; and declared industry sponsorship or conflicts of interest. Although a formal risk-of-bias instrument was not applied, methodological completeness was considered narratively when interpreting the evidence and was observed to vary systematically by study design. Randomized and randomized-derived reports (*n* = 10) generally provided structured long-term assessment, including a priori responder definitions, explicit attrition accounting, and itemized device-related outcomes. In contrast, the 55 observational reports were heterogeneous in these respects: prospective cohorts and registries tended to report attrition and device events more consistently than retrospective single-center series, and a priori responder definitions were more common in contemporary 10 kHz, closed-loop, and painful diabetic neuropathy reports than in older mixed neuropathic pain cohorts. Opioid-outcome reporting was the most heterogeneous descriptor across all study designs and is summarized separately in Supplementary Table S5. This descriptive stratification informed the synthesis but did not enter a formal certainty-of-evidence framework.

### 2.9. Synthesis of Results

Because of the marked heterogeneity in indications, technologies, study designs, outcome definitions, and follow-up structures, no quantitative meta-analysis was attempted. Instead, the results were synthesized descriptively using both numerical summary and narrative mapping approaches, consistent with scoping review methodology [14,15,16].

The evidence was summarized across four complementary axes: (1) study design and maturity of evidence, including randomized and randomized-derived long-term reports; (2) clinical population, including FBSS/PSPS, chronic back and leg pain, painful diabetic neuropathy, nonsurgical refractory back pain, complex regional pain syndrome, and mixed neuropathic pain cohorts; (3) stimulation modality, including conventional tonic or mixed/unspecified SCS, high-frequency SCS, burst-related reports, and physiologic closed-loop SCS; and (4) outcome domain, including pain durability, function/disability, quality of life, opioid use, and device-related revision/explantation/complication burden.

The final narrative synthesis aimed to identify where long-term evidence was most consistent, where it remained sparse or heterogeneous, and which methodological weaknesses most strongly limited the interpretation of long-term SCS effectiveness.

## 3. Results

### 3.1. Study Selection

The staged study selection process is summarized in Figure 1. The PubMed/MEDLINE search yielded 3188 records and the Scopus search yielded 3678 records, for a total of 6866 records before reconciliation. After pragmatic pre-screening, sequential title/abstract screening, iterative full-text retrieval, and reconciliation of overlapping publications, 292 unique full-text reports were assessed for eligibility. Of these, 202 were excluded after full-text review, most commonly because they did not provide extractable outcomes beyond 24 months, used a non-eligible outcome framework, or represented duplicate cohort reporting without a distinct long-term contribution. The final evidence map and narrative synthesis comprised 65 unique reports with extractable SCS outcomes at ≥24 months.

### 3.2. Overall Characteristics of the Included Evidence Base

The final evidence base comprised 65 unique reports representing a cumulative report-level population of 11,518 participants. This number should not be interpreted as the number of unique patients, because several mature trials generated more than one publication. The long-term literature was methodologically heterogeneous but remained dominated by non-randomized evidence. Ten reports were classified as randomized or randomized-derived long-term reports, whereas the remaining 55 reports were observational in nature. To avoid non-mutually exclusive design labels, reports were grouped at a higher level according to whether they arose from randomized clinical programs or from observational clinical datasets; overall characteristics of the included evidence base are summarized in Table 1. The remaining reports comprised prospective and retrospective cohorts, registries, case-series–type clinical follow-up reports, and long-term observational extensions of implanted patient cohorts [1,2,3,10,12,13].

The included reports spanned several eras of SCS practice, from earlier conventional SCS series in neuropathic limb pain and failed back surgery syndrome to more recent long-term studies in painful diabetic neuropathy, nonsurgical refractory back pain, and physiologic closed-loop stimulation [1,2,3,4,5,6,7,12,13]. This temporal breadth increased the relevance of the evidence map, but also introduced substantial variation in hardware generation, stimulation paradigms, and outcome-reporting standards.

### 3.3. Clinical Populations Represented

The largest indication category was mixed chronic pain (30/65 reports; 46.2%), followed by FBSS/PSPS (16/65; 24.6%), chronic back and/or leg pain (6/65; 9.2%), CRPS (5/65; 7.7%), and painful diabetic neuropathy (4/65; 6.2%). The remaining reports represented smaller categories, including mixed neuropathic pain, nonsurgical refractory back pain, intractable leg pain, and refractory limb pain. The distribution of included reports by indication, together with representative longest follow-up ranges for the main indication groups, is summarized in Table 2A.

Where report-level detail permitted, the internal composition of this mixed category was explored further and is summarized in Supplementary Table S4. Within the mixed chronic pain category, the most frequently co-occurring patient groups were failed back surgery syndrome or persistent spinal pain combined with other neuropathic limb pain conditions, reflecting the real-world composition of multidisciplinary pain programs from which many of the older cohort reports originated. A smaller subset of reports within this category represented mixed neuropathic populations without a dominant spinal pain component, including post-herpetic neuralgia, phantom limb pain, and peripheral ischemic pain. The proportion of reports explicitly identifying FBSS or PSPS as a primary or major subgroup within mixed categories was higher than the FBSS/PSPS-specific counts alone suggest, further reinforcing the spinal pain anchoring of the long-term SCS evidence base. The modern long-term evidence in painful diabetic neuropathy and nonsurgical refractory back pain was narrower in volume but methodologically more contemporary and more standardized in endpoint reporting [6,7,12].

### 3.4. Stimulation Modalities Represented

Most included reports were categorized as conventional/mixed/unspecified SCS (47/65; 72.3%). More modality-specific clusters included 10 kHz/high-frequency SCS (7 reports), paddle-lead SCS (3 reports), and ECAP-controlled closed-loop SCS (3 reports). The remaining reports represented smaller modality categories, including multicolumn or other specialized long-term SCS configurations. The distribution of reports by stimulation modality, together with representative longest follow-up ranges for the principal modality categories, is shown in Table 2B.

This distribution indicates that the long-term evidence base is still dominated by conventional or mixed/unspecified SCS practice, especially in older reports [1,2,10,11]. In contrast, waveform-specific and platform-specific long-term data were concentrated in more recent publications, particularly for 10 kHz SCS and closed-loop SCS, where endpoint structure and reporting quality were generally more explicit [3,4,5,6,7,12,13]. Accordingly, the literature captures both the historical durability of SCS as a therapeutic class and the emerging long-term performance of contemporary systems, but these strata should not be interpreted as methodologically equivalent.

### 3.5. Outcome Domains Reported Beyond 24 Months

The most frequently reported long-term domain was pain durability (60/65 reports; 92.3%), followed by function and/or health-related quality of life (57/65; 87.7%), opioid-related outcomes (55/65; 84.6%), and device-related revision/explantation/complication outcomes (53/65; 81.5%) (Table 2C). Across the included literature, pain outcomes were usually presented as maintained pain-score improvement, responder persistence, continued clinically meaningful benefit, or ongoing device use associated with pain control [1,3,5,6,7,8,9,10,11,12,13]. Functional and quality-of-life outcomes included disability scales, generic health-status instruments, patient global impression measures, and, in a smaller subset, sleep-related endpoints [2,3,6,7,12].

Opioid-related reporting was notably heterogeneous. Of the 55 reports that addressed opioid-related outcomes, the majority (approximately 60–65%) reported opioid-related findings in qualitative or semi-quantitative terms only, including statements of reduction, cessation, or decreased analgesic burden without a standardized quantitative framework [8,9,10,11,12]. A minority of reports (approximately 35–40%) employed structured medication quantification approaches, such as morphine milligram equivalents (MMEs) or comparable numerical metrics, enabling at least partial cross-study comparison. The full distribution of opioid-reporting formats across the included reports is provided in Supplementary Table S5. The predominance of qualitative formats limits cross-study comparability and weakens the interpretation of opioid reduction as a durable secondary marker of treatment success.

Device-related outcomes were also inconsistently defined, but many reports included information on revision, explantation, lead migration, infection, hardware failure, or discontinuation due to loss of efficacy or intolerance [1,2,8,9,10,11]. Across the evidence base, these device-maintenance outcomes often accompanied otherwise favorable long-term pain findings, underscoring that durable benefit and device burden frequently coexist.

### 3.6. Direction of the Long-Term Effectiveness Signal

At a broad descriptive level, the long-term literature more often supported persistence of benefit than complete erosion of effect, although the magnitude and consistency of that signal differed across clinical populations and stimulation platforms. In longstanding FBSS/PSPS and chronic back/leg pain cohorts, multiple mature reports described sustained pain relief, continued device use, and ongoing patient-reported benefit beyond 24 months, including evidence arising from both classic randomized trials and long-term follow-up studies [1,2,10,11,12,13]. However, these same bodies of evidence also documented revision burden, later treatment failure, and non-trivial explantation in a subset of implanted patients, indicating that long-term benefit was common but not universal [8,9,10,11].

A relatively coherent contemporary signal was observed in painful diabetic neuropathy, where long-term 10 kHz SCS reports consistently described maintained improvements in pain and quality of life beyond 24 months [12]. In nonsurgical refractory back pain, available long-term reports likewise suggested that a clinically meaningful proportion of patients maintained benefit beyond 24 months, particularly in high-frequency SCS cohorts [6,7]. The painful diabetic neuropathy literature also appeared to include probable overlap between publications derived from the same underlying clinical program, and this should be considered when interpreting the apparent weight of evidence for this subgroup.

For ECAP-controlled closed-loop SCS, randomized and randomized-derived long-term reports indicated sustained multidomain benefit, including pain, function, and broader patient-centered outcomes [3,13]. These data are clinically important because they extend beyond pain intensity alone, but the long-term closed-loop evidence base remains numerically limited and includes two reports derived from the same EVOKE cohort.

### 3.7. Randomized and Randomized-Derived Long-Term Evidence

Long-term randomized evidence was present but remained limited in number relative to the overall evidence base. The randomized and randomized-derived reports are presented explicitly in Table 3, with each row linked to the corresponding reference, study population, modality, sample size, longest reported follow-up, and notes where economic analyses or probable cohort overlap require specific interpretive caution. These reports arose primarily from three domains: classic FBSS trials, more recent high-frequency SCS programs, and the EVOKE closed-loop platform [1,2,3,10,11,12,13,21,22].

These reports were especially important because they provided stronger internal validity and more structured longitudinal assessment than most observational datasets. At the same time, not all randomized-derived publications represented standalone primary long-term trial reports. Some were follow-up extensions, secondary analyses, or domain-specific re-analyses of already established trial cohorts. Thus, randomized evidence strengthened the evidence map, but did not eliminate the broader heterogeneity of endpoint definitions, report structure, and cohort overlap within the long-term SCS literature [2,3,10,11,12,13].

### 3.8. Evidence Gaps Identified by the Long-Term Evidence Map

To make the impact of cohort overlap more concrete, included reports were examined for shared clinical programs on the basis of trial name, recruitment period, indication, sample size, stimulation modality, and author group. Three principal overlapping clusters were identified within the final evidence base. First, the EVOKE program of ECAP-controlled closed-loop SCS contributed two long-term reports derived from the same underlying randomized cohort (Mekhail et al., 2023 [13]; Kapural et al., 2023 [3]). Second, the SENZA-PDN program of 10 kHz SCS for painful diabetic neuropathy contributed two or three closely related long-term reports drawing on the same trial population (Petersen et al., 2023 [21]; Petersen et al., 2026 [12]; Argoff et al., 2025 [22]). Third, classic FBSS randomized trials contributed multiple linked publications combining clinical outcomes and cost-utility analyses on the same patient cohorts (North et al., 2005 [1]; North et al., 2007 [10]). When these overlapping clusters were taken into account, the 65 included reports corresponded to approximately 58–60 distinct clinical cohorts rather than 65 independent patient populations. Cohort relationships are flagged explicitly in Table 3 and in Supplementary Table S3, where each linked publication is annotated with its primary cohort identifier.

Several recurring evidence gaps emerged from the mapped literature. First, cohort overlap remained common in mature clinical programs, especially in highly cited randomized and multicenter studies, complicating interpretation of cumulative participant numbers and the apparent weight of evidence [2,3,10,12,13]. Second, long-term endpoint definitions were inconsistent, particularly for responder status, maintained benefit, opioid reduction, and treatment discontinuation. Third, mixed chronic pain and mixed/unspecified SCS categories still represented a large proportion of the literature, limiting the precision of indication-specific and technology-specific conclusions.

Taken together, the evidence map showed that long-term SCS reporting is broad but unevenly distributed. The strongest long-term clusters were observed in FBSS/PSPS and related spinal pain populations, with smaller but increasingly important contemporary clusters in painful diabetic neuropathy, nonsurgical refractory back pain, and closed-loop SCS [1,2,3,6,7,10,11,12,13]. The outcome domains most often captured beyond 24 months were pain durability, function/quality of life, opioid-related outcomes, and device-related maintenance outcomes.

## 4. Discussion

This scoping review mapped the clinical literature reporting SCS outcomes at 24 months or longer and identified a long-term evidence base that is broader than is often assumed, yet substantially less coherent than the short-term literature may suggest. Across 65 unique reports with extractable long-term outcomes, the dominant signal was not one of uniform or permanent treatment success, but rather of sustained benefit in a substantial proportion of appropriately selected patients, accompanied by persistent heterogeneity in outcome definitions, cohort structure, and device-related maintenance burden [1,2,3,6,7,10,11,12,13]. In practical terms, the literature beyond 24 months supports the view that SCS can provide durable clinical benefit, but it also makes clear that durability is conditional, indication-dependent, and inseparable from revision, reprogramming, explantation, and attrition over time [1,2,8,9,10,11,12].

One of the clearest findings of this review is that the long-term SCS literature remains anchored in spinal pain populations, especially FBSS/PSPS and chronic back/leg pain cohorts, where the evidence base includes both foundational randomized trials and mature observational follow-up studies burden [1,2,10,11,12,13]. These studies consistently suggest that benefit can persist well beyond the early implant period, but they also show that the long-term course of therapy is dynamic rather than static. In these cohorts, the clinically relevant endpoint is not simply whether pain scores improve shortly after implantation, but whether meaningful benefit persists in the face of disease chronicity, device-related events, therapeutic adaptation, and changing patient expectations [1,2,8,9,10,11,12]. This distinction matters because it aligns the interpretation of SCS with the real logic of longitudinal pain care rather than with the narrower logic of early efficacy assessment alone.

The review also highlights an important shift in the contemporary evidence base. Although older long-term studies established the principle that SCS could remain effective beyond 2 years, more recent reports in painful diabetic neuropathy, nonsurgical refractory back pain, and physiologic closed-loop SCS provide a more structured and methodologically mature account of long-term benefit [3,6,7,12,13]. In painful diabetic neuropathy, the long-term signal appears relatively coherent, with sustained improvements in pain and health-related quality of life reported beyond 24 months in modern high-frequency SCS cohorts [12]. Similarly, in nonsurgical refractory back pain, recent high-frequency SCS studies suggest that durable benefit is not restricted to post-surgical spinal pain populations, but may also extend to carefully selected non-surgical cohorts [6,7].

The long-term evidence for ECAP-controlled closed-loop SCS deserves separate comment. The available randomized and randomized-derived reports indicate sustained multidomain benefit, including pain, function, and broader patient-centered outcomes, beyond 24 months [3,13]. This is clinically important because it shifts the discussion away from pain intensity alone and toward a more integrated interpretation of treatment durability. At the same time, the numerical size of the long-term closed-loop evidence base remains modest relative to the wider historical literature in conventional or mixed SCS. Moreover, two of the three long-term closed-loop reports identified in the present review arose from the EVOKE program and may reflect the same underlying cohort analyzed from different perspectives [3,13]. Closed-loop SCS should therefore be viewed not as definitively established superior long-term therapy across all indications, but as one of the most methodologically promising contemporary strands within the field [3,13].

One of the most important conceptual contributions of the present review is to show how strongly the apparent strength of the literature depends on how long-term effectiveness is defined. In some reports, durability was represented by maintenance of mean pain reduction. In others, it was reflected by responder persistence, continued device use, patient global improvement, or absence of explantation due to loss of efficacy [1,2,8,9,10,11,12]. These are related but not equivalent constructs. A patient may remain implanted despite only partial benefit, while another may achieve good pain relief but limited functional recovery, and a third may reduce opioid consumption without meeting a conventional responder threshold. Long-term SCS effectiveness is therefore inherently multidimensional, and the literature becomes difficult to compare when studies privilege different endpoints without standardization [3,8,9].

Building on this observation, the present review proposes an operational framework for reporting long-term SCS effectiveness that future trials and cohort studies could adopt to enhance cross-study comparability. The framework defines five minimum reporting domains for any outcome assessment at ≥24 months. (1) Pain durability should be reported as both maintained mean change from baseline on a standardized pain scale and the proportion of patients meeting an a priori responder definition (e.g., ≥50% reduction in pain intensity or the minimum clinically important difference, with the chosen threshold prespecified). (2) Function and health-related quality of life should be reported using at least one validated patient-reported instrument (e.g., ODI, EQ-5D, PROMIS, or condition-appropriate equivalent) with both baseline and long-term values and the corresponding within-patient change. (3) Opioid-related outcomes should be reported using a formally quantified metric (MEDD or MME) rather than qualitative reduction statements or binary cessation alone, with the analytic population clearly defined. (4) Device-related outcomes should include explicit, denominator-anchored rates of revision, explanation, lead migration, infection, and discontinuation due to loss of efficacy, separating events occurring before and after 24 months where feasible. (5) Treatment persistence should be reported as the proportion of originally implanted patients with an active and clinically effective SCS device at the long-term time point, alongside the proportion lost to follow-up. Reporting these five domains in a consistent, denominator-anchored way would substantially reduce the heterogeneity that currently limits comparative interpretation across long-term SCS studies and would align the SCS literature with established outcome reporting standards in chronic pain trials [20].

This problem is particularly visible in the reporting of opioid-related outcomes. Many long-term SCS reports referred to opioid reduction, cessation, or lower medication burden, but the underlying reporting framework was highly inconsistent (Supplementary Table S5). [8,9,10,11,12]. Some studies used structured medication quantification or morphine-equivalent measures, whereas others provided only qualitative reduction statements or binary cessation outcomes. This lack of standardization limits cross-study comparability and weakens the interpretation of opioid reduction as a durable secondary marker of treatment success. Given the increasing clinical and policy relevance of opioid-sparing strategies in chronic pain care, future long-term SCS studies should report opioid outcomes using reproducible and explicitly defined metrics alongside pain, function, quality of life, and device-maintenance endpoints [8,9,10,11,12]. In this respect, the current literature still treats opioid outcomes too often as ancillary observations rather than as rigorously measured longitudinal endpoints.

The economic implications of these findings are equally important. SCS is a therapy with substantial upfront cost, and its value proposition depends not simply on whether benefit can be achieved, but on whether benefit can be maintained long enough to justify implantation and follow-up costs [10,11]. The two key economic studies identified in this review support that principle in specific settings: SCS was less expensive and more effective than reoperation in FBSS over a mean 3.1-year follow-up, and 10 kHz SCS was cost-saving and cost-effective versus low-frequency SCS in a model-based evaluation over a longer time horizon [10,11]. These findings do not justify broad claims that all SCS is uniformly cost-effective across all technologies and indications. They do, however, reinforce the central argument of the present review: long-term durability is not a secondary methodological preference, but a core determinant of the clinical and economic meaning of SCS [10,11].

Several limitations of the underlying evidence base constrain interpretation. First, the literature is heavily affected by cohort overlap, particularly in mature trial programs and multicenter clinical initiatives, where several publications may arise from the same patient population [2,3,10,12,13]. This makes cumulative patient counts inherently vulnerable to inflation if interpreted naively. For that reason, the participant total in this review was reported at the report level, not as a unique-patient denominator. Second, the dominance of broad categories such as mixed chronic pain and conventional/mixed/unspecified SCS reflects real-world practice but weakens analytic precision. These categories are useful for evidence mapping, but they are poor substitutes for tightly phenotyped clinical populations or clearly specified technologies [8,9,26]. Third, older reports often lack the granularity expected in contemporary long-term neuromodulation research, particularly with respect to programming strategy, attrition handling, explantation reporting, and multidomain outcomes [1,2,4,5,6].

The review itself also has limitations. Although the project was ultimately structured and reported according to PRISMA-ScR principles, the workflow retained a staged, practice-based character, including pragmatic pre-screening, iterative full-text acquisition, and post hoc reconciliation of long-term reports. In particular, the initial reduction from 6866 retrieved records to a manually reviewed title/abstract set of 604 records was based on a pragmatic pre-screening step rather than a prospectively specified filtering protocol. Although this step was intended to remove clearly irrelevant records, it may have excluded some potentially eligible studies before formal screening and therefore limits the claim of exhaustive retrieval. In addition, no formal risk-of-bias tool or GRADE certainty framework was applied because the objective was evidence mapping rather than comparative effect estimation [14,15,16,17]. These decisions are methodologically defensible within scoping review practice, but they mean that the present review should not be interpreted as a formal certainty-ranked effectiveness synthesis.

The main implication for future research is not simply that more long-term SCS studies are needed, but that better long-term SCS studies are needed. Future work should prioritize: (1) clear indication-specific cohorts rather than broad mixed pain categories; (2) explicit reporting of stimulation modality and programming strategy; (3) standardized multidomain outcome sets including pain, function, quality of life, opioid use, revision, and explantation; (4) transparent handling of reprogramming and retreatment as part of the therapeutic pathway rather than as peripheral events; and (5) better distinction between unique cohort reports and secondary analyses of the same population [3,8,9,12,13]. In a field where therapeutic durability is central to both patient expectations and reimbursement logic, long-term evidence must mature beyond isolated extension reports toward reproducible, methodologically explicit longitudinal research.

**Figure 2:**
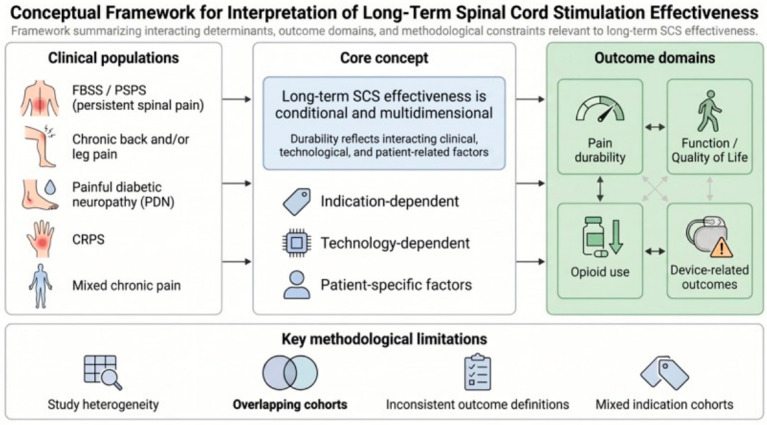
Conceptual framework for interpretation of long-term spinal cord stimulation effectiveness.

Taken together, the present evidence map supports a balanced conclusion. The literature beyond 24 months does not justify an unqualified claim that SCS provides universally durable benefit across all chronic pain populations and all stimulation technologies. However, it does show that sustained effectiveness is a plausible and repeatedly observed outcome in several clinically important settings, particularly in spinal pain populations, painful diabetic neuropathy, and selected contemporary modality-specific programs [1,2,3,6,7,10,11,12,13]. The enduring challenge for the field is therefore no longer whether long-term benefit is possible, but how to define it consistently, measure it rigorously, and predict it with enough precision to improve patient selection and long-term care pathways (Figure 2).

The framework summarizes interacting determinants, multidomain outcome measures, and key methodological constraints relevant to the interpretation of spinal cord stimulation (SCS) outcomes beyond 24 months. The left panel illustrates the principal clinical populations represented in the long-term SCS literature, including failed back surgery syndrome/persistent spinal pain syndrome (FBSS/PSPS), chronic back and/or leg pain, painful diabetic neuropathy (PDN), complex regional pain syndrome (CRPS), and mixed chronic pain cohorts. The central panel emphasizes that long-term SCS effectiveness is conditional and multidimensional, shaped by indication-dependent, technology-dependent, and patient-specific factors. The right panel presents the principal outcome domains assessed in long-term studies, including pain durability, function and quality of life, opioid-related outcomes, and device-related outcomes. The bottom panel highlights major methodological limitations affecting interpretation of the evidence base, including study heterogeneity, overlapping cohorts, inconsistent outcome definitions, and mixed indication cohorts. Created with BioRender.com.

## 5. Conclusions

In this PRISMA-ScR-informed scoping review, the literature reporting SCS outcomes at 24 months or longer supported the view that durable benefit is achievable in a substantial subset of patients, particularly in persistent spinal pain/FBSS-related populations, and in more contemporary evidence, in painful diabetic neuropathy, nonsurgical refractory back pain, and selected closed-loop SCS cohorts [1,2,3,6,7,10,11,12,13]. However, the long-term evidence base was heterogeneous in design, indication mix, stimulation modality, and outcome definitions, and it remained strongly affected by cohort overlap, variable report maturity, and inconsistent reporting of revision, explantation, and opioid-related outcomes [2,3,8,9,10,11,12,13].

These findings suggest that the key challenge in long-term SCS research is no longer to establish whether sustained benefit is possible, but to determine in whom, under which technological and clinical conditions, and by what outcome framework such benefit should be judged [3,8,9,12]. Long-term effectiveness should not be reduced to pain intensity alone. It should be understood as a multidimensional construct that includes durability of analgesia, functional recovery, health-related quality of life, treatment persistence, device-maintenance burden, and where relevant, opioid reduction [2,3,8,9,12].

Accordingly, future long-term SCS studies should prioritize indication-specific cohorts, standardized multidomain outcome reporting, transparent handling of reprogramming and retreatment, and clearer separation of unique cohorts from secondary analyses of the same clinical populations [3,8,9,12,13]. Until such evidence matures further, the current literature can be interpreted as supporting plausible and repeatedly observed long-term effectiveness in three clinically important settings: spinal pain populations (particularly FBSS/PSPS), where both randomized and mature observational evidence consistently suggests sustained benefit beyond 24 months; painful diabetic neuropathy, where contemporary high-frequency SCS reports provide a more structured, endpoint-standardized signal of durable analgesia and quality-of-life improvement; and physiologic closed-loop SCS, where emerging randomized evidence indicates multidomain benefit at 24–36 months. Across all other populations and technologies, the evidence base does not yet support a certainty-ranked effectiveness estimate and requires further prospective, indication-specific investigation.

## Figures and Tables

**Table 1:** Overall characteristics of the included long-term evidence base.

Characteristic	Value
Unique reports included in final synthesis	65
Report-level population	11,518
Randomized/randomized-derived reports	10
Observational reports	55
Most frequent indication category	Mixed chronic pain (30/65; 46.2%)
Most frequent modality category	Conventional/mixed/unspecified SCS (47/65; 72.3%)
Main long-term domains most often reported	Pain durability; function/quality of life; opioid-related outcomes; revision/explantation/complications

SCS, spinal cord stimulation.

**Table 2:** (**A**) Distribution of included reports by clinical population, with representative ranges of the longest reported follow-up within the major indication categories. (**B**) Distribution of included reports by stimulation modality, with representative ranges of the longest reported follow-up within the major modality categories. (**C**) Frequency of long-term outcome domains reported beyond 24 months.

**(A)**		
**Clinical Population**	**Reports, *n* (%)**	**Representative Range of Longest Follow-Up, Months**
Mixed chronic pain	30 (46.2%)	24–240
FBSS/PSPS	16 (24.6%)	24–127.7
Chronic back and/or leg pain	6 (9.2%)	24–48
CRPS	5 (7.7%)	35.6–120
Painful diabetic neuropathy	4 (6.2%)	24–84
Other populations	4 (6.2%)	24–60
**(B)**		
**Stimulation modality**	**Reports, *n* (%)**	**Representative range of longest follow-up, months**
Conventional/mixed/unspecified SCS	47 (72.3%)	24–240
10 kHz/high-frequency SCS	7 (10.8%)	24–36
Paddle-lead SCS	3 (4.6%)	24–79.7
ECAP-controlled closed-loop SCS	3 (4.6%)	24–36
Other specialized configurations	5 (7.7%)	24–48
**(C)**		
**Outcome domain**		**Reports, *n* (%)**
Pain durability		60 (92.3%)
Function and/or health-related quality of life		57 (87.7%)
Opioid-related outcomes		55 (84.6%)
Revision/explantation/complication outcomes		53 (81.5%)

FBSS, failed back surgery syndrome; PSPS, persistent spinal pain syndrome; CRPS, complex regional pain syndrome. SCS, spinal cord stimulation; ECAP, evoked compound action potential.

**Table 3:** Randomized and randomized-derived reports included in the long-term evidence map, with linked references, study population, stimulation modality, sample size, longest reported follow-up, and notes on cohort relationship where relevant.

Report (Short Citation)	Population (*n*)	Modality	Longest Follow-Up, Months	Notes
North et al., 2005 [1]	Persistent/recurrent radicular pain after lumbosacral surgery (FBSS-enriched spinal pain) (50)	Conventional SCS vs. reoperation	36	Primary randomized comparison
Kumar et al., 2008 [2]	Neuropathic pain/FBSS-enriched multicenter population (100)	Conventional SCS	24	Randomized trial follow-up
North et al., 2007 [10]	FBSS/PSPS (60)	Conventional SCS vs. reoperation	37	Economic analysis of randomized trial data; primary endpoint was cost-utility rather than clinical long-term SCS outcomes
North et al., 2005 [23]	FBSS/PSPS (24)	Percutaneous vs. laminectomy electrodes	35	Randomized electrode-design trial
Kemler et al., 2008 [24]	CRPS type I (54)	Conventional SCS	60	Five-year randomized-trial follow-up
Kapural et al., 2016 [25]	Chronic back and leg pain (198)	10 kHz SCS vs. low-frequency SCS	24	SENZA-RCT 24-month report
Petersen et al., 2023 [21]	Painful diabetic neuropathy (113)	10 kHz SCS	24	Randomized-trial follow-up; probable cohort relationship with Petersen et al., 2026 [12]
Argoff et al., 2025 [22]	Painful diabetic neuropathy (216)	10 kHz SCS	24	Randomized-derived sensory secondary analysis
Mekhail et al., 2023 [13]	Chronic trunk and/or limb pain/back and leg pain (134)	ECAP-controlled closed-loop vs. open-loop SCS	36	EVOKE primary long-term trial report
Kapural et al., 2023 [3]	Chronic trunk and/or limb pain/back and leg pain (134)	ECAP-controlled closed-loop vs. open-loop SCS	24	EVOKE multidomain secondary analysis; probable cohort overlap with Mekhail et al., 2023 [13]

SCS, spinal cord stimulation; FBSS, failed back surgery syndrome; PSPS, persistent spinal pain syndrome; CRPS, complex regional pain syndrome; ECAP, evoked compound action potential; RCT, randomized controlled trial; SENZA-RCT, Senza Randomized Controlled Trial (10 kHz SCS vs. conventional SCS).

## Data Availability

The data supporting the findings of this review are contained within the article and its Supplementary Materials. Additional working files used during staged screening and data charting are available from the corresponding author upon reasonable request.

## Supplementary Materials

The following supporting information can be downloaded at: https://www.mdpi.com/article/10.3390/jcm15103939/s1. Supplementary Table S1. Full electronic search strategies; Supplementary Table S2. Detailed full-text exclusion categories; Supplementary Table S2A. Pre-screening exclusion breakdown for the reduction from retrieved records to records retained for formal title/abstract review, with operational exclusion definitions and approximate counts per category; Supplementary Table S3. Included reports with extractable outcomes at ≥24 months; Supplementary Table S4. Internal composition of the mixed chronic pain category where report-level detail permitted; Supplementary Table S5. Formats of opioid-outcome reporting across included reports; and the PRISMA-ScR checklist.

## Abbreviations

The following abbreviations are used in this manuscript:

SCS	Spinal cord stimulation
FBSS	Failed back surgery syndrome
PSPS	Persistent spinal pain syndrome
CRPS	Complex regional pain syndrome
PDN	Painful diabetic neuropathy
ECAP	Evoked compound action potential
JBI	Joanna Briggs Institute
PRISMA-ScR	Preferred Reporting Items for Systematic reviews and Meta-Analyses extension for Scoping Reviews
HRQoL	Health-related quality of life
MME	Morphine milligram equivalent
MEDD	Morphine equivalent daily dose
RCT	Randomized controlled trial
SENZA-RCT	Senza Randomized Controlled Trial (10 kHz SCS versus conventional SCS)
GRADE	Grading of Recommendations, Assessment, Development and Evaluations
DBS	Deep brain stimulation
PNS	Peripheral nerve stimulation
tDCS	Transcranial direct current stimulation
MeSH	Medical Subject Headings
DOI	Digital object identifier
