# Supplementary material for: Long-Term Effectiveness of Spinal Cord Stimulation Beyond 24 Months: A PRISMA-ScR-Informed Scoping Review
Source: J Clin Med. 2026 May 20;15(10):3939. doi: 10.3390/jcm15103939 (PMC13208038; doi:10.3390/jcm15103939)
Supplement: Supplementary file 1 [file jcm-15-03939-s001.zip › PRISMA_ScR_Checklist.pdf]

PRISMA-ScR Checklist

Page references correspond to the final rendered manuscript.

Section	PRISMA-ScR item	Location/Page(s)
Title	Identify the report as a scoping review	Title page
Abstract	Structured summary	Abstract, pp. 1–2
Introduction	Rationale and objectives	Section 1, pp. 2–3
Methods	Protocol and registration	Section 2.1, p. 4
Methods	Eligibility criteria	Section 2.2, pp. 4–5
Methods	Information sources and search	Sections 2.3–2.4; Supplementary Table S1, pp. 5–6
Methods	Selection of sources of evidence	Sections 2.4–2.5; Figure 1; Supplementary Table S2, pp. 5–6
Methods	Data charting	Section 2.7, p. 7
Methods	Critical appraisal	Section 2.8, p. 7
Methods	Synthesis of results	Section 2.9, p. 7
Results	Selection of sources of evidence	Section 3.1; Figure 1, p. 8
Results	Characteristics of sources of evidence	Sections 3.2–3.4; Tables 1–3, pp. 8–11
Results	Synthesis of charted results	Sections 3.5–3.8, pp. 10–12
Discussion	Summary of evidence and limitations	Section 4, pp. 12–15
Funding	Sources of funding	Funding statement, p. 16
