# Supplementary material for: Long-Term Effectiveness of Spinal Cord Stimulation Beyond 24 Months: A PRISMA-ScR-Informed Scoping Review
Source: J Clin Med. 2026 May 20;15(10):3939. doi: 10.3390/jcm15103939 (PMC13208038; doi:10.3390/jcm15103939)
Supplement: Supplementary file 1 [file jcm-15-03939-s001.zip › Supplementary_materials_revised.pdf]

Supplementary Materials

Long-Term Effectiveness of Spinal Cord Stimulation Beyond 24 Months: A PRISMA-ScR–Informed Scoping Review

Supplementary Tables S1, S2, S2A, S3–S5 are provided as a separate Excel file (JCM_supplementary_tables_submission_ready.xlsx). The PRISMA-ScR checklist is provided as a separate Word document (JCM_PRISMA_ScR_checklist_submission_ready.docx).

Supplementary Table S1. Full electronic search strategies.

Supplementary Table S2. Detailed categories of full-text exclusion after assessment of 292 unique full-text reports.

Supplementary Table S2A. Pre-screening exclusion breakdown for the reduction from 6866 retrieved records to 604 records retained for formal title/abstract review, with operational exclusion definitions and approximate counts per category.

Supplementary Table S3. Included reports with extractable spinal cord stimulation outcomes at ≥24 months (n = 65).

Supplementary Table S4. Internal composition of the mixed chronic pain category where report-level detail permitted.

Supplementary Table S5. Formats of opioid-outcome reporting across included reports (n = 55 reports addressing opioid outcomes).

The PRISMA-ScR checklist is provided as a separate submission document.

Supplementary Method. Retrospectively Defined Relevance-Based Pre-Screening Framework

Before formal title/abstract screening, a retrospectively defined relevance-based pre-screening framework was applied to remove records considered clearly outside the scope of the review on the basis of exported bibliographic information. Records were excluded at this stage only when title/abstract export data indicated that they were unequivocally: (1) non-clinical record types, including reviews, editorials, commentaries, conference abstracts without full-text availability, and protocol-only papers; (2) non-original publication types; (3) outside the intervention scope of spinal cord stimulation, including non-SCS neuromodulation modalities with no SCS arm, pharmacological or surgical studies without SCS component, and basic science or animal studies; or (4) unrelated to long-term clinical outcomes in chronic pain populations, including paediatric populations, non-pain indications, or follow-up clearly below 24 months when explicitly stated in the title or abstract. The largest proportion of records excluded at the pre-screening stage fell into categories (1) and (2). When relevance could not be confidently excluded at the export-screening

stage, records were retained for formal title/abstract review. Because this framework was defined retrospectively rather than prospectively protocolized, the possibility that some potentially eligible studies were excluded before formal screening cannot be fully eliminated and is acknowledged as a methodological limitation of the present review.
